# Editorial: “The Role of Immune Checkpoint Molecules in Solid and Hematopoietic Stem Cell Transplantation”

**DOI:** 10.3389/fimmu.2021.822558

**Published:** 2021-12-20

**Authors:** Lambros Kordelas, Frans H. J. Claas, Vera Rebmann

**Affiliations:** ^1^ Department of Hematology and Stem Cell Transplantation, University Hospital Essen, Essen, Germany; ^2^ Leiden University Medical Center, Leiden, Netherlands; ^3^ Institute for Transfusion Medicine, University Hospital Essen, Essen, Germany

**Keywords:** immune checkpoint molecules, hematopoietic stem cell transplantation, solid transplantation, tolerance, HLA

The success of both solid organ transplantation (SOT) and hematopoietic stem cell transplantation (HSCT) requires down-regulation of the allo-immune response. SOT and HSCT could only become standard therapies for many end-stage organ diseases or severe haematological malignancies thanks to the development of modern strategies, which aim at the suppression of the activity of T cells, B cells and NK cells recognizing genetic disparities between donor and recipient and as an ultimal goal the induction of tolerance. A proper downregulation of the alloimmune response is a prerequisite to prevent acute or chronic graft rejection in SOT and to avoid graft-versus-host disease (GvHD) in HSCT. However, keeping an equilibrium between a sufficient suppression of the allo-immune response or tolerance for the allo-antigens and maintenance of an adequate immune defense against infections and relapse continues to be a major challenge in transplantation.

Against this background, the role of immune checkpoints molecules (ICM) is of superior interest. Activation of ICM by interaction of co-inhibitory receptors with its cognate ligands is essential for maintaining immune homeostasis, diminishing tissue damage, and preventing unwanted autoimmunity. Dysregulation of ICM molecules can result in immune escape from host immune defense in infection and cancer. To further delineate these crucial pathways, a Research Topic was dedicated on *“The Role of Immune Checkpoint Molecules in Solid and Hematopoietic Stem Cell Transplantation”*.

Köhler et al. review the importance of ICM for relapse after allogeneic HSCT. They summarize that immune checkpoint blockade can increase anti-tumor immunity, but has been primarily successful in solid cancer therapy and Hodgkin lymphoma so far. Relapse after allogeneic HSCT is mainly thought to be attributable to loss of the graft-versus-leukemia (GVL) effect. One potential mechanism of immune escape from the GVL effect is the inhibition of allogeneic T cells *via* engagement of inhibitory receptors on their surface including PD-1, CTLA-4, TIM3, and others. This review provides an overview of current evidence for a role of immune checkpoint molecules for relapse and its treatment after allogeneic HSCT. The retrospective study of Mytilineos et al. analyses the influence of expression levels and mismatch permissiveness on the HLA-DPB1 degree of compatibility in the context of allogeneic HSCT. HLA-DPB1 mismatches can be classified in *permissive* and *non-permissive* mismatches by T-cell epitope matching. Non-permissive HLA-DPB1 mismatches showed significantly increased aGvHD risk if they were accompanied by two HLA-DPB1 mismatches in GvH direction or one mismatched highly expressed patient allotype. Non-permissive HLA-DPB1 mismatches is associated with a significantly higher risk of acute GvHD and non-relapse mortality. This study suggests that DP non-permissiveness associated with two HLA-DPB1 mismatches or at least on highly expressed mismatched patient allotype should be avoided. Kordelas et al. investigate the clinical significance of soluble PD-1 (sPD-1) after allogeneic HSCT regarding GvHD, relapse, and overall survival (OS) in a mono-centric cohort of 82 patients. They observed that low sPD-1 plasma levels at month one, two or three post HSCT were associated with acute GvHD grade III-IV, the onset of moderate/severe chronic GvHD and inferior OS, DFS, and TRM, respectively. Hence, this study pinpoints the soluble inhibitory co-receptor PD-1 as a promising candidate molecule for the prediction of clinical HSCT outcome. Chen et al. investigate whether the single-nucleotide polymorphisms (SNPs) of the co-stimulatory genes within non-HLA regions were related to the outcomes of allogeneic HSCT. Their results revealed that nine SNPs in the CTLA4 gene, five SNPs in the PDCD1 gene, two SNPs in the TNFSF4 gene, and four SNPs in the CD28 gene were significantly associated with the adverse outcomes following allogeneic HSCT. Duygu et al. review how NK cell alloreactivity and anti-viral immunity are regulated by NK cell receptors belonging to the KIR family and interacting with classical HLA class I molecules, or by NKG2A/C and LILRB1/KIR2DL4 engaging non-classical HLA-E or -G. Specifically, the authors focus on how NK cells contribute to the allo-immune response upon kidney transplantation either by promoting allograft rejection through lysis of cells of the transplanted organ or by promoting alloreactive T cells. Zhang et al. analyse the potential role of the novel immunosuppressant Belatacept for prevention of rejection following kidney transplant. To test the hypothesis that Belatacept combined with BTLA overexpression, may effectively attenuate acute rejection after kidney transplantation, the authors used a rat kidney transplantation model comparing graft rejection in single and combined therapy. By means of immunohistochemistry and flow cytometry, antigen-stimulated immune response by mixed lymphocyte culture, western blot and qRT-PCR analyses, the authors could show that Belatacept combined with BTLA overexpression attenuates acute rejection after kidney transplantation and prolonged kidney graft survival.

As up to now the key area of focus in ICM research and clinical implication has been in the field of cancer, this Research Topic highlights the contribution of ICM to allograft tolerance and to clinical outcome of SOT as well as to allogeneic HSCT.

## Author Contributions

All authors listed have made a substantial, direct, and intellectual contribution to the work and approved it for publication.

## Conflict of Interest

The authors declare that the research was conducted in the absence of any commercial or financial relationships that could be construed as a potential conflict of interest.

## Publisher’s Note

All claims expressed in this article are solely those of the authors and do not necessarily represent those of their affiliated organizations, or those of the publisher, the editors and the reviewers. Any product that may be evaluated in this article, or claim that may be made by its manufacturer, is not guaranteed or endorsed by the publisher.

